# Racial and Ethnic Disparities in the Prevalence of Stress and Worry, Mental Health Conditions, and Increased Substance Use Among Adults During the COVID-19 Pandemic — United States, April and May 2020

**DOI:** 10.15585/mmwr.mm7005a3

**Published:** 2021-02-05

**Authors:** Lela R. McKnight-Eily, Catherine A. Okoro, Tara W. Strine, Jorge Verlenden, NaTasha D. Hollis, Rashid Njai, Elizabeth W. Mitchell, Amy Board, Richard Puddy, Craig Thomas

**Affiliations:** ^1^CDC COVID-19 Social and Behavioral Health Team; ^2^Division of Human Development and Disability, National Center on Birth Defects and Developmental Disabilities, CDC; ^3^CDC COVID-19 Response Team.

In 2019, approximately 51 million U.S. adults aged ≥18 years reported any mental illness,* and 7.7% reported a past-year substance use disorder^†^ (*1*). Although reported prevalence estimates of certain mental disorders, substance use, or substance use disorders are not generally higher among racial and ethnic minority groups, persons in these groups are often less likely to receive treatment services (*1*). Persistent systemic social inequities and discrimination related to living conditions and work environments, which contribute to disparities in underlying medical conditions, can further compound health problems faced by members of racial and ethnic minority groups during the coronavirus disease 2019 (COVID-19) pandemic and worsen stress and associated mental health concerns (*2**,**3*). In April and May 2020, opt-in Internet panel surveys of English-speaking U.S. adults aged ≥18 years were conducted to assess the prevalence of self-reported mental health conditions and initiation of or increases in substance use to cope with stress, psychosocial stressors, and social determinants of health. Combined prevalence estimates of current depression, initiating or increasing substance use, and suicidal thoughts/ideation were 28.6%, 18.2%, and 8.4%, respectively. Hispanic/Latino (Hispanic) adults reported a higher prevalence of psychosocial stress related to not having enough food or stable housing than did adults in other racial and ethnic groups. These estimates highlight the importance of population-level and tailored interventions for mental health promotion and mental illness prevention, substance use prevention, screening and treatment services, and increased provision of resources to address social determinants of health. How Right Now (Qué Hacer Ahora) is an evidence-based and culturally appropriate communications campaign designed to promote and strengthen the emotional well-being and resiliency of populations adversely affected by COVID-19–related stress, grief, and loss (*4*).

CDC licensed results from Porter Novelli’s PN View 360, a nationwide, weekly opt-in Internet panel survey of U.S. adults. The survey was administered by ENGINE Insights in English to U.S. adults aged ≥18 years using the Lucid platform (*5*); respondents who had not taken a survey in the previous 20 waves of survey administration were eligible to participate. Quota sampling was conducted by ENGINE Insights to identify respondents, and statistical weighting was used during the analysis to match proportions in the 2019 Current Population Survey; therefore, the sample was representative of the overall U.S. population by sex, age, region, race/ethnicity, and education. CDC licensed the results of the PN View 360 survey after data collection from Porter Novelli. This activity was reviewed by CDC and was conducted consistent with applicable federal law and CDC policy.^§^ In both April and May, 502 respondents participated, for a combined total of 1,004 respondents; the survey included questions about increases in or initiation of substance use during the COVID-19 pandemic,^¶^ symptoms of current depression,^**^ and suicidal thoughts/ideation,^††^ as well as questions about psychosocial stress (e.g., feeling isolated and alone), stigma or discrimination (from being blamed for spreading COVID-19), and social determinants of health (e.g., food instability). Combined and weighted response percentages and 95% confidence intervals (CIs) were calculated by using PROC SURVEYFREQ in SAS statistical software (version 9.4; SAS Institute). Because respondents were recruited from an opt-in panel rather than by probability sampling, other than using CIs, no inferential statistical tests were performed.^§§^

The overall prevalence estimates of current depression, suicidal thoughts/ideation, and initiation of or increase in substance use were 28.6%, 8.4%, and 18.2%, respectively (Table). Symptoms of current depression were reported 59% more frequently by Hispanic adults (40.3%) than by non-Hispanic White (White) persons (25.3%). Estimates of self-reported suicidal thoughts/ideation among Hispanic persons (22.9%) were four times those among non-Hispanic Black (Black) persons (5.2%) and White persons (5.3%) and approximately twice those of multiracial and non-Hispanic persons of other races/ethnicities (8.9%).^¶¶^ Increased or newly initiated substance use was reported among 36.9% of Hispanic respondents, compared with 14.3%–15.6% among all other respondents.

**TABLE:** Weighted prevalence estimates of current depression,* suicidal thoughts/ideation,^†^ and substance use increase or initiation^§^ among adults aged ≥18 years, by race/ethnicity — Porter Novelli View 360 survey, United States, April and May 2020

Race/Ethnicity	Unweighted no. of persons	Weighted % (95% CI)		
		Current depression	Suicidal thoughts/Ideation	Substance use increase or initiation
**Total**	**1,004**	**28.6 (25.6–31.5)**	**8.4 (6.6–10.2)**	**18.2 (15.7–20.7)**
White, NH	657	25.3 (21.9–28.7)	5.3 (3.6–6.9)	14.3 (11.6–17.0)
Black, NH	100	27.7 (18.7–36.7)	5.2 (0.7–9.7)	15.6 (8.4–22.7)
Hispanic/Latino	118	40.3 (31.3–49.3)	22.9 (15.2–30.6)	36.9 (28.1–45.7)
Other, NH^¶^	129	31.4 (22.8–40.0)	8.9 (3.6–14.1)	15.1 (8.4–21.7)

**Abbreviations:** CI = confidence interval; DSM-IV = *Diagnostic and Statistical Manual of Mental Disorders, Fourth Edition;* NH = non-Hispanic/Latino.

* Defined as a score of ≥10 on the eight-item Patient Health Questionnaire (PHQ-8). The PHQ-8 is adapted from the nine-item PHQ (PHQ-9), which is based on the nine criteria for diagnosis of depressive disorders in the DSM-IV.

^†^ Defined as an affirmative response to the question “At any time in the past 30 days, did you seriously think about trying to kill yourself?”

^§^ Defined as an affirmative response to the question “Have you started or increased using substances to help you cope with stress or emotions during the COVID-19 pandemic? Substance use includes alcohol, legal or illegal drugs, or prescriptions drugs that are taken in a way not recommended by your doctor.”

^¶^ Includes participants who identified as Native American/Alaska Native, Asian, multiracial, or another race/ethnicity.

Among U.S. adults overall, sources of psychosocial stress included family health (36.3%), feelings of isolation or loneliness (28.6%), worry about getting ill from COVID-19 or infecting others (25.7%), worry about the death of a loved one or persons dying (15.2%), workplace COVID-19 exposure (13.5%), and stigma or discrimination from being blamed for spreading COVID-19 (4.1%) (Figure 1). White adults were more likely to report stress and worry about the health of family members and loved ones (39.3%) than were Black adults (24.5%). A larger percentage of multiracial and non-Hispanic adults of other races/ethnicities reported stress and worry about stigma or discrimination associated with being blamed for spreading COVID-19 (12.9%) than did White (2.4%) or Hispanic (3.7%) adults.

**FIGURE 1:**
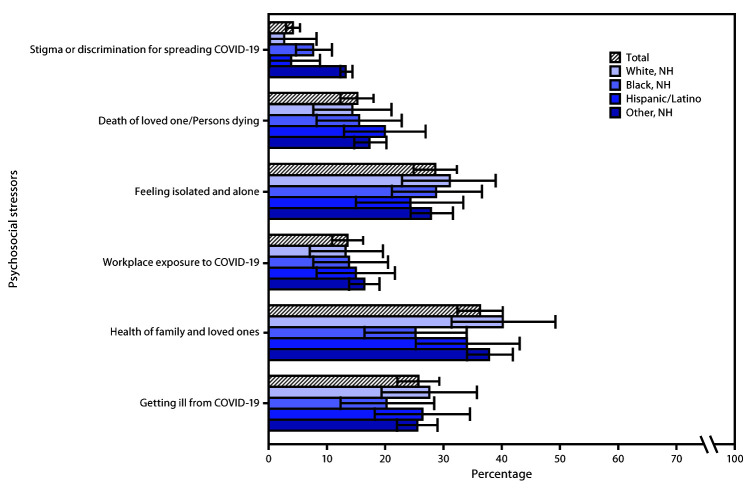
Weighted prevalence estimates* of self-reported stress and worry about psychosocial stressors among adults aged ≥18 years (N = 1,004), overall and by race/ethnicity^†^ — Porter Novelli View 360 survey, United States, April and May 2020 **Abbreviations:** COVID-19 = coronavirus disease 2019; NH = non-Hispanic/Latino. * With 95% confidence intervals shown by error bars. ^†^ Other non-Hispanic minority groups include participants who identified as Native American/Alaska Native, Asian, multiracial, or another race/ethnicity.

Estimates of stress and worry about social determinants of health included possible job loss (27.1%), ability to obtain needed health care (18.4%), not having enough food (14.4%), and housing instability (11.8%) (Figure 2). A higher percentage of Hispanic adults reported stress about not having enough food (22.7%) or stable housing (20.7%) than did White adults (11.9% and 9.2%, respectively).

**FIGURE 2:**
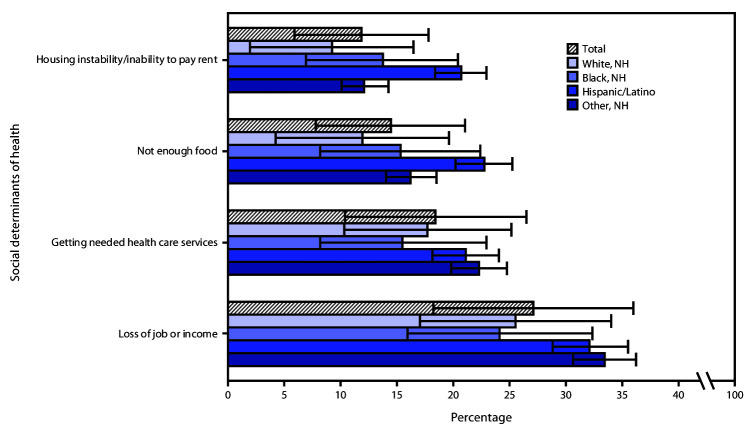
Weighted prevalence estimates* of self-reported stress and worry about social determinants of health among adults aged ≥18 years (N = 1,004), overall and by race/ethnicity**†** — Porter Novelli View 360 survey, United States, April and May 2020 **Abbreviations:** COVID-19 = coronavirus disease 2019; NH = non-Hispanic/Latino. * With 95% confidence intervals shown by error bars. ^†^ Other non-Hispanic minority groups include participants who identified as Native American/Alaska Native, Asian, multiracial, or another race/ethnicity.

## Discussion

Selected mental health conditions and initiation of or increase in substance use to cope with stress or emotions during the COVID-19 pandemic were commonly reported by U.S. adults responding to an opt-in survey in April and May 2020. The prevalence of current depression, suicidal thoughts/ideation, and increased or newly initiated substance use was also higher for some racial and ethnic minority groups, especially Hispanic respondents. Hispanic adults reported higher levels of stress and worry about not having enough food or stable housing than did White adults.

A review of baseline mental health data from other national surveys, which used different study designs and methodologies, suggests potential increases in the mental health outcomes included in this report. Current depression among adults aged ≥18 years was estimated to be 7.0% by the 2019 National Health Interview Survey (*6*) and 23.5% by the 2020 Household Pulse Survey during April 23–May 5, 2020,*** compared with an estimated 28.6% of adults aged ≥18 years in this report. In the 2019 National Survey on Drug Use and Health, 4.8% of U.S. adults aged ≥18 years reported serious suicidal thoughts (*1*), whereas 8.4% of adults in this report indicated having suicidal thoughts/ideation. Recent data from another U.S. panel survey indicated that 40.9% of respondents aged ≥18 years reported mental or behavioral health concerns during the COVID-19 pandemic, with 13.3% of respondents reporting that they increased or initiated substance use (*7*), compared with nearly 20% of respondents in this report.

In 2019, not having enough food was reported three times more frequently by Black persons and two times more frequently by Hispanic persons than by White persons (*8*). Stigma, including harassment and discrimination, combined with social or structural determinants of health, such as inadequate access to safe housing, healthy food, transportation, and health care, can increase the risk for chronic stress among persons in racial and ethnic minority groups and potentially affect their mental and physical health, including contributing to poor outcomes from COVID-19 (*3*,*4*,*7*). Additional evidence-based measures to promote population-level mental health in adults are important,^†††^ including screening for mental illness (e.g., depression) (*9*) and substance misuse (e.g., alcohol misuse) (*10*). Persons identified by screening as having a higher risk for mental illness are best served when treated or referred to a health care provider for intervention, including counseling, referral to services, or treatment (*9*,*10*). Because a substantial proportion of mental health care occurs in primary care settings,^§§§^ health care access is important for addressing mental health and substance use conditions, including opioid use. Although racial and ethnic minority group members did not report more psychosocial stress related to health care access than did White persons, disparities in access to health care, including having a usual source of care, are preexisting factors that affect physical and mental health.^¶¶¶^

Additional public health measures are critical to address the mental and behavioral health consequences of the COVID-19 pandemic. How Right Now (Qué Hacer Ahora) is a communications campaign designed to promote and strengthen the emotional well-being and resiliency of populations adversely affected by COVID-19–related stress, grief, and loss. The campaign offers evidence-based and culturally appropriate information and resources to address the emotional health needs of adults in both English and Spanish (*4*). CDC is working with national, tribal, state, and community partners; academic institutions; and other federal agencies to define, measure, and improve the emotional well-being and quality of life of the U.S. population across the lifespan. Additional resources are available from CDC.**** Behavioral health and addiction services resources are available through a free Substance Abuse and Mental Health Services Administration’s Disaster Distress Helpline (1-800-985-5990)^††††^ and addiction treatment locators.^§§§§^

The findings in this report are subject to at least five limitations. First, all responses were self-reported and might be subject to recall, response, or social desirability biases. Second, although survey responses were weighted to be representative of U.S. population demographics, whether responses in this opt-in panel sample are representative of the broader U.S. population and which biases might have affected the findings are not known. Third, the generalizability of estimates for Hispanic populations was limited because the survey was administered in English on the Internet; therefore, Spanish-only speakers might not have been included. This report suggests that additional studies are needed, and consideration of surveys that focus on sampling Hispanic/Latino populations who speak Spanish might be helpful. Fourth, the data are cross-sectional, which precludes the ability to make causal inferences. Finally, the sample size was small (1,004), which limited certain types of analysis and resulted in small cell sizes for some comparisons.

Addressing barriers or disruptions to access to and delivery of mental health and substance use services during the COVID-19 pandemic, including considerations for health care systems, practices, and providers using telehealth coverage^¶¶¶¶^; consideration of parity in insurance coverage for mental health and substance use services; and use of virtual mental health treatment and substance use recovery groups, is important. Policies and structural programs can be adapted or developed to reduce preexisting racial and ethnic group disparities in social determinants of health (e.g., housing,***** food, access to health care, and income security) while also addressing psychosocial stressors unique to communities with large racial and ethnic minority populations. The mental health and psychosocial needs of U.S. adults, including persons in racial and ethnic minority groups, are an important consideration when promoting community resilience and preserving access to and provision of services during the COVID-19 pandemic.

**Summary**

**What is already known about this topic?**

Racial and ethnic minority groups have experienced disparities in mental health and substance misuse related to access to care, psychosocial stress, and social determinants of health.

**What is added by this report?**

Combined prevalence estimates of current depression, initiating or increasing substance use, and suicidal thoughts/ideation among U.S. adults aged ≥18 years were 28.6%, 18.2%, and 8.4%, respectively. Hispanic adults reported a higher prevalence of psychosocial stress related to not having enough food or stable housing than did adults in other racial and ethnic groups.

**What are the implications for public health practice?**

Addressing psychosocial stressors, mental health conditions, and substance misuse among U.S. adults during the COVID-19 pandemic is important, as are interventions tailored for racial and ethnic minority groups.

## Footnotes

* Adults with any mental illness were defined as those having any mental, behavioral, or emotional disorder in the past year that met specified criteria from the *Diagnostic and Statistical Manual of Mental Disorders*, *Fourth Edition,* (DSM-IV) (excluding developmental disorders and substance use disorders).

† Persons who met the criteria for dependence or abuse for alcohol or illicit drugs in the past 12 months based on criteria specified in the DSM-IV were defined as having a substance use disorder.

§ 45 C.F.R. part 4601[b][2].

¶ Substance use initiation or increase was defined as an affirmative response to the question “Have you started or increased using substances to help you cope with stress or emotions during the COVID-19 pandemic? Substance use includes alcohol, legal or illegal drugs, or prescriptions drugs that are taken in a way not recommended by your doctor.”

** Current depression was defined as a score of ≥10 on the eight-item Patient Health Questionnaire (PHQ-8). The PHQ-8 is adapted from the nine-item PHQ (PHQ-9), which is based on the nine criteria for diagnosis of depressive disorders in the DSM-IV.

†† Having suicidal thoughts/ideation was defined as an affirmative response to the question “At any time in the past 30 days, did you seriously think about trying to kill yourself?”

§§ The standard errors assume that the weighted estimates used in the Taylor series linearization are approximately unbiased or at the very least are consistent. This assumption of approximate unbiasedness is based on the assertion that any differences between the survey sample and the target population on key survey outcomes are corrected by the weighting. No analysis was conducted to validate that assertion. https://www.aapor.org/AAPOR_Main/media/MainSiteFiles/NPS_TF_Report_Final_7_revised_FNL_6_22_13.pdf

¶¶ Other non-Hispanic minority groups included participants who identified as Native American/Alaska Native, Asian, multiracial, or another race/ethnicity.

*** https://www.cdc.gov/nchs/covid19/pulse/mental-health.htm

†††
https://www.cdc.gov/coronavirus/2019-ncov/daily-life-coping/managing-stress-anxiety.html

§§§
https://www.cdc.gov/nchs/products/databriefs/db311.htm#ref3

¶¶¶
https://www.ahrq.gov/research/findings/nhqrdr/nhqdr18/index.html

**** https://www.cdc.gov/populationhealth/well-being

††††
https://www.samhsa.gov/find-help/disaster-distress-helpline

§§§§
https://www.samhsa.gov/medication-assisted-treatment/practitioner-program-data/treatment-practitioner-locator

¶¶¶¶
https://www.cdc.gov/coronavirus/2019-ncov/hcp/telehealth.html

***** https://www.jchs.harvard.edu/sites/default/files/sonhr14-color-full_0.pdf
